# Efficient planning and implementation of optimal foraging strategies under energetic constraints

**DOI:** 10.1093/pnasnexus/pgag009

**Published:** 2026-01-17

**Authors:** Yipei Guo, Ann M Hermundstad

**Affiliations:** Janelia Research Campus, Howard Hughes Medical Institute, Ashburn, VA 20147, USA; Janelia Research Campus, Howard Hughes Medical Institute, Ashburn, VA 20147, USA

**Keywords:** local search, exploration–exploitation tradeoff, decision-making, uncertain environments, network computation

## Abstract

To successfully forage, animals must balance the energetic cost of searching for food sources with the energetic benefit of exploiting those sources. While the Marginal Value Theorem provides one normative account of this balance by specifying that a forager should leave a food patch when its energetic yield falls below the average yield of other patches in the environment, it assumes the presence of other readily reachable patches. In natural settings, however, a forager does not know whether it will encounter additional food patches, and it must balance potential energetic costs and benefits accordingly. Upon first encountering a patch of food, it faces a decision of whether and when to leave the patch in search of better options, and when to return if no better options are found. Here, we explore how this search should be structured when the existence of other food patches is unknown, and when searching for them requires energy that can only be harvested from a single known food patch. We identify conditions under which it is more favorable to explore the environment in several successive trips rather than in one long exploration, and we show how the optimal sequence of trips depends on the forager’s beliefs about the environment. This optimal strategy is well approximated by a local decision rule that can be implemented by a simple neural circuit architecture. Together, this work highlights how energetic constraints and prior beliefs shape optimal foraging strategies, and how such strategies can be approximated by simple neural networks.

Significance Statement Foraging animals must balance the potential benefits of discovering valuable resources with the energetic costs of exploration. Existing theoretical frameworks describe the decision to leave a known resource in favor of other options, assuming those options exist and can be found through random search. However, in uncertain environments, a key question arises: how should the forager structure the search process itself to ensure that it maintains energy reserves if better resources are not found? We derive the optimal search strategy in such settings, and find that the structure and timing of successive search bouts depends critically on the forager’s prior expectations about the environment. Near-optimal performance can be achieved through a local decision rule implemented in a biologically plausible architecture.

## Introduction

Searching for new food sources is energetically costly. Successful foragers must balance this energetic cost against the gains of exploiting known food sources. The Marginal Value Theorem (MVT) provides a normative framework for describing this trade-off (1, 2). In its original formulation, food is assumed to be distributed across discrete patches, and travel between patches incurs a fixed time cost. Due to resource depletion, a forager gains energy at a diminishing rate while exploiting a patch. The optimal policy, derived by maximizing the long-term average rate of energy intake, prescribes that a forager should leave a patch when its energetic yield falls below the average yield of other patches in the environment (1). Crucially, this approach assumes that the forager will reliably encounter another patch after each departure.

This basic framework has been extended in numerous ways to account for additional ecological and behavioral factors. Some models consider uncertainty about patch quality (3, 4), or incorporate movement decisions such as how fast to travel between patches (5). Other studies relax the assumption of fixed travel times by modeling inter-patch search as a stochastic search process (6, 7), and such mechanistic models have been used to examine how the type of stochastic process affects patch-finding success (8–10). Yet, across all these model extensions, a key assumption typically remains: that other food patches exist and will eventually be found.

In natural environments, however, the existence of additional food sources is often unknown, and a forager must weigh the energetic costs of unsuccessful search against the potential benefits of discovering new patches. Many insects, including flies, have been observed to engage in local exploratory search around a known food source (11–13), and the average duration of their exploration increases over time (14). This strategy—of first sampling the nearby environment before expanding outward if no alternatives are found—may be an efficient way to conserve energy while maintaining access to a reliable food source. Alternative strategies—such as beginning with a more extensive exploration of the environment in the hope of quickly identifying better food sources—carry a higher risk of energetic depletion, but may offer long-term benefits by enabling more efficient foraging later on. These different possibilities raise a fundamental question about optimal foraging: upon first encountering a patch of food, how should a forager structure its search for new patches when (i) the existence of those patches is not known with certainty and (ii) searching for those patches requires energy that can only be harvested from a single known food patch?

Here, we explore optimal strategies for structuring search under such energetic constraints. Because the forager faces the possibility that there will not be any food patches nearby, we assume that it initiates a search only when, and for as long as, it has enough energy to reliably return to a known food patch. In these settings, we show that it can be advantageous to partition the search into multiple successive trips, depending on the forager’s belief about the distribution and content of patches in the environment. Although the optimal search strategy often requires planning multiple trips into the future, we find that a local decision rule can closely approximate optimal performance and is implementable in a biologically plausible circuit architecture. Together, this work provides insights into how foragers should best balance energetic costs and demands when searching for food in uncertain environments, and how this energetic balance can be achieved at the level of neural circuits and behavior.

## Results

### General setup

**Fig. 1.**
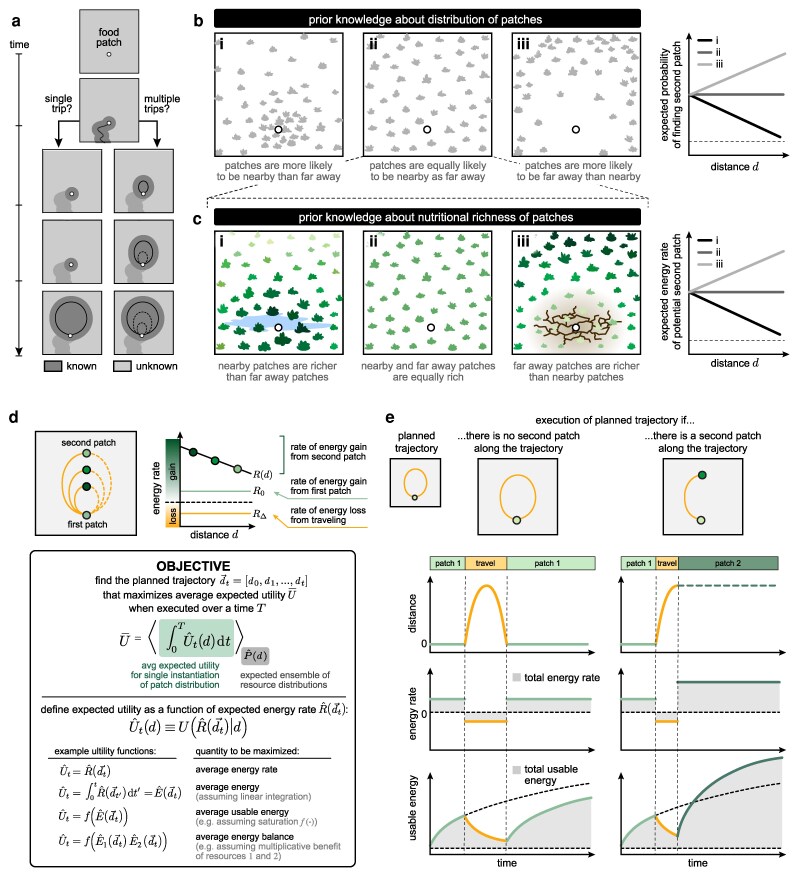
An energetically constrained forager faces a decision of how often and how far to travel. a) A forager on a food patch must decide whether to explore and in how many trips. b, c) The forager maintains knowledge about the spatial distribution (b) and nutritional content (c) of food patches. Here and below, we use darker saturation to indicate patches with higher nutritional content. d) We consider a 1D scenario in which the forager gains energy at a rate $R_{0}$ while harvesting from a known food patch, loses energy at a rate $R_{\Delta }$ while traveling, and can gain additional energy at a rate R(d) if a second food patch is found at a distance *d* from the original food spot. Box: To weigh the potential costs and benefits of traveling away from the first food patch, we measure the average expected utility $\left\langle \int_{0}^{T}{\hat{U}}_{t}({\vec{d}}_{t}\;|\;d)dt\right\rangle$ of executing a planned trajectory ${\vec{d}}_{t}$, averaged over all possible locations of the closest food patch *d*. *U* can take on many forms, depending on the specific demands of the forager. e) The forager seeks the trajectory that maximizes the average expected utility over time and across possible distributions of patches. In planning this trajectory, the forager must account for the possibilities that it does (right column) or does not (left column) encounter a second patch along its trajectory.

We consider a hungry forager that has just discovered a patch of food in a new environment (Fig. 1a). After harvesting some food from the patch, the forager must decide whether to continue harvesting from the same patch, or whether to leave the patch and seek out better options. If there are no additional patches in its vicinity, the forager might be best served by harvesting as much as possible from the patch in order to gain enough energy to make a long trip to another environment. However, if there are nearby patches with higher yields or complementary resources, the forager would benefit from more quickly seeking out and exploiting these nearby patches.

Without knowing which of these situations it faces, the forager must use its prior belief about the distribution and nutritional content of food patches in the environment to decide whether it is beneficial to search, and if beneficial, how far to travel. Because the forager faces the possibility that there will not be any food patches nearby, we assume that it structures its search in a series of outbound and inbound trips to ensure that it returns to the known food patch if it fails to find a second patch. The forager must then decide whether to search the environment in a single long trip, or whether to break the search into multiple trips (Fig. 1a). The optimal number and sequence of such trips will depend on the forager’s prior beliefs about the environment, and it will be constrained by how much energy the forager can harvest from the original food spot.

To guide its decisions about whether and when to leave the first food patch, we assume that the forager maintains prior beliefs about the distribution of food patches that it is likely to encounter in a new environment (Fig. 1b) and the nutritional content of those food patches (Fig. 1c). We assume that the belief about these two environmental features is maintained in two separate functions that depend only on distance from the forager: (i) the expected probability $\hat{P}(d)$ of finding a second food patch at a distance *d* away, and (ii) the expected rate of energetic return per unit time, $\hat{R}(d)$, from potential patches at that distance. The shape of these two functions determines the relative probability and energetic return of finding food patches that are nearby vs. far away. For example, if the expected probability of finding a second food patch decays with distance, the forager expects that the first food patch is indicative that other food patches are more likely to be clustered nearby rather than far away. Analogously, if the expected energy rate of a second food patch decays with distance, the forager expects that nearby food patches will either have higher nutritional yield or will be more nutritionally diverse than distant patches. As we will show, the interaction between these two functions will shape the forager’s optimal search strategy.

We assume that the forager’s decisions are guided by an energy reserve that grows over time as the forager feeds, and depletes over time as the forager travels. The forager must thus decide how to allocate its feeding and traveling decisions to maintain this energy reserve, analogous to classic formulations of foraging (1). We further assume that this energy reserve cannot drop below zero at any time. As a result, the total energy in the reserve acts as a hard constraint on the maximum distance that the forager can travel before returning to the patch to replenish the reserve. Thus, in order to decide whether to make an exploratory trip, the forager must weigh the expected energetic gain from finding a second patch along the outbound trip (minus the expected energetic cost of outbound travel) against the expected energetic cost of traveling the full outbound and inbound trip, should no second patch be found (Fig. 1d). We assume that the forager harvests energy at a fixed rate $R_{0}>0$ while on the first food patch and expends energy at a fixed rate $R_{\Delta }<0$ while traveling; in natural settings, these parameters would be determined by factors such as the nutritional value of resources at the current patch, the roughness of the terrain, and the forager’s basal metabolic rate. To weigh the potential costs and benefits of traveling away from the first patch, we assume that the forager uses its prior beliefs to plan a trajectory that consists of one or more successive outbound and inbound trips, subject to the constraint that the forager’s energy reserve cannot drop below zero at any point along the trajectory.

To compare different planned trajectories, we evaluate the expected utility of each trajectory over a total time *T*. Because the forager only maintains a belief about the expected *distribution* of possible patches $\hat{P}(d)$, but not about the specific instantiation of that distribution, we compute the average expected utility over all possible instantiations of $\hat{P}(d)$. For each instantiation, we assume that the forager would carry out its planned trajectory unless or until it encounters a second patch, at which point it would harvest energy at an average rate $R(d^{†})$ that is determined by the location $d^{†}$ of the second patch (in this way, R(d) can represent the instantaneous energy rate of the second patch or the average rate of traveling between and exploiting two complementary patches). We define the expected utility $\hat{U}$ to be a function of the expected energy rate $\hat{R}(d)$. Different functional forms of $\hat{U}$ correspond to different quantities that the forager might aim to maximize, such as the average rate or amount of usable energy harvested from a single patch, or the average energetic balance between different types of patches (Fig. 1d).

In what follows, we explore how the forager’s belief about the distribution and quality of food patches impacts its planned foraging trajectory. For tractability, we consider a simplified 1D scenario in which the forager travels at a constant velocity v=1 when traveling away from and returning back to a known food patch at the origin, d=0, within an environment of size *D* (Fig. 1e). In this case, a planned trajectory consists of a sequence $\{d{\}}_{i}^{N}$ of *N* outbound trips to and return trips from locations $d_{1},d_{2},\ldots ,d_{N}$ initiated at times $t_{1},t_{2},\ldots ,t_{N}$. We take food patches to be distributed with fixed probability P(d)=p. When the forager encounters a second patch, we assume that it can harvest energy at a rate R(d)=μ(1−γd). We take $\mu >R_{0}$, such that there is a potential benefit to exploration, and γ≥0, such that there is no benefit from further exploration once a second patch is found. Taken together, these assumptions mean that when evaluating the expected utility of a foraging trajectory, we need only consider the possible locations of the *nearest* patch, which follows $P_{1}(d)=pe^{-pd}$ for a uniform distribution P(d)=p. We evaluate trajectories with respect to the utility function U=R (SI Section 1), which corresponds to maximizing the average energy rate over a time interval *T* (or, equivalently, the final energy at time *T*). Under this assumption, for a planned outbound trip to location *d*, the forager should leave the food patch as soon as it has enough energy to travel the outbound and return distance 2d, such that the time spent on the food patch before a trip to *d* is given by $\Omega (d)=2d|R_{\Delta }|/R_{0}$ (SI Section 1).

To derive the optimal trajectories for maximizing expected utility, we assume that the forager knows the rates $R_{0}$ and $R_{\Delta }$. We further assume that the forager begins with prior beliefs ${\hat{P}}_{1}(d)=pe^{-pd}$ and $\hat{R}(d)=\mu (1-\gamma d)$ (i.e. the forager knows the correct form and parameters of the distribution of food patches in the environment, but does not know the specific location of the nearest patch). We first consider cases where these beliefs are fixed in time and are used to plan and execute a sequence of trips. We then consider the case where the forager can update its belief ${\hat{P}}_{1}(d)$ about the nearest patch, and can use this updated belief to re-evaluate a planned sequence of trips.

### Moderately dense and nutritionally rich environments encourage multiple trips

**Fig. 2.**
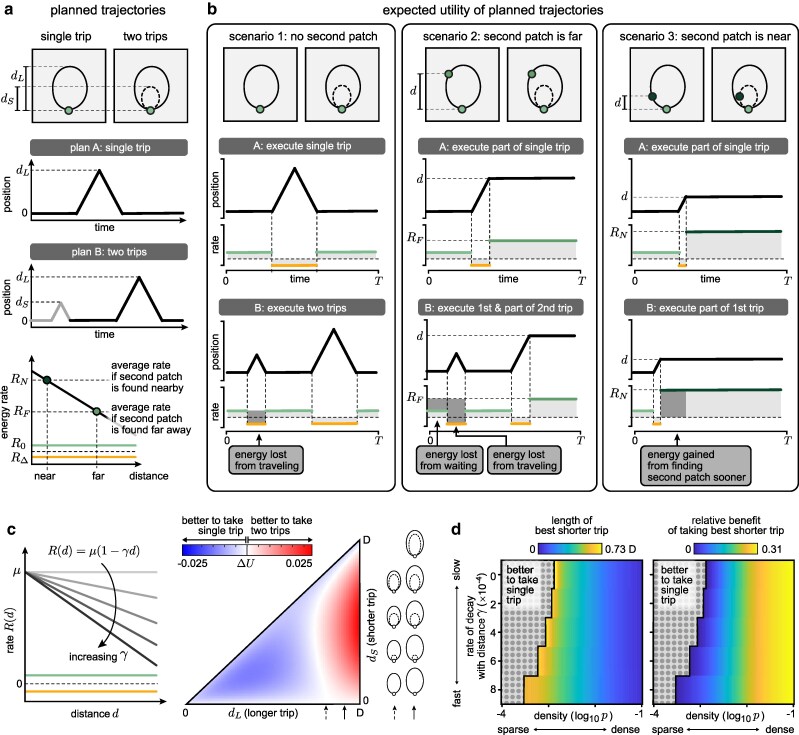
In certain environments, it can be advantageous to explore in multiple trips. a) Given a single trip of length $d_{L}$, we evaluate the relative gain in utility of first taking a shorter trip of length $d_{S}$. As in Fig. 1, we use darker saturation to indicate patches with higher nutritional content that yield higher energy rates. b) The relative gain of taking a short trip before a long trip can be decomposed into three different contributions: (i) the energy lost from the short trip if no second patch is found (left column), (ii) the energy lost from waiting and traveling if the second patch is beyond the reach of the shorter trip (middle column), and (iii) the energy gained from finding the second patch sooner if it is within reach of the shorter trip (right column). c) We use *γ* to parameterize the rate of energy gain R(d) from patches a distance *d* away (left panel). For a given value of *γ*, we can determine the relative gain in utility ΔU of taking a short trip of length $d_{S}$ before a longer trip of length $d_{L}$ (right panel). We select the best combination of short and long trips for which ΔU>0; if ΔU<0, the agent should use a single trip to explore the environment. d) As *p* increases and as *R* decays more rapidly with distance (i.e. *γ* increases), it becomes more beneficial (right panel) to take an increasing short earlier trip (left panel). The following parameters were used in (c) and (d): D=1000, $R_{0}=0.1$, $R_{\Delta }=-0.1$, μ=10, T=8000; in (c): γ=0.001, p=0.24771.

We began by exploring whether, and under what conditions, it would be better for the forager to plan to explore the environment in two successive trips vs. one long trip (Fig. 2a). We first determined the optimal single trip to and from a location $d_{L}$ that would maximize the expected utility over a time interval *T*, given a fixed set of environmental parameters *p*, *γ*, and *μ*. We then determined the scenarios under which it would have been favorable to first plan a shorter trip to and from a location $d_{S}$.

The change in expected utility from having an additional first trip, ΔU, depends on the forager’s expectation of encountering a second food patch. This expectation can be decomposed into three different scenarios that occur with different probabilities, depending on the density of patches *p*:

**Scenario 1: No second patch.** If there is no second patch within a distance $d_{L}$, it would be detrimental to first take a short trip because the forager would lose additional energy $\left(R_{0}-R_{\Delta }\right)$ while traveling the distance $2d_{S}$ (relative to using the same time to harvest from the first patch at a rate $R_{0}$). This scenario occurs with probability $e^{-pd_{L}}$, and leads to an expected *loss* in utility (Fig. 2b, left column):
  (1) $$\Delta \langle \bar{U}\rangle_{S1}T=-e^{-pd_{L}}2d_{S}(R_{0}-R_{\Delta })$$
**Scenario 2: Second patch is far.** If there is a second patch at a distance $d>d_{S}$, it would again be detrimental to first take a short trip. This is not only because the forager would lose additional energy $\left(R(d)-R_{\Delta }\right)$ while traveling the distance $2d_{S}$ (relative to using the same time to harvest from the second patch at a rate R(d)), but also because in preparing to take the first trip, the forager would lose additional energy $\Omega (d_{S})(R(d)-R_{0})$ as a result of harvesting from the first patch (relative to using the same time to harvest from the second patch at a rate R(d)). When averaged over all possible locations $d_{S}<d<d_{L}$ of the second patch, each occurring with probability $P_{1}(d)$, this leads to an expected *loss* in utility (Fig. 2b, middle column):
  (2) $$\Delta \langle \bar{U}\rangle_{S2}T=-\int_{d_{S}}^{d_{L}}P_{1}(d)(2d_{S}(R(d)-R_{\Delta })+2\frac{\left|R_{\Delta }\right|}{R_{0}}d_{S}(R(d)-R_{0}))\;dd.$$
**Scenario 3: Second patch is near.** If there is a second patch within a distance $d_{S}$, it would be beneficial to first take a short trip because the forager could find and exploit the second patch more quickly, thereby gaining additional energy $\left(\Omega (d_{L})-\Omega (d_{S}))(R(d)-R_{0}\right)$ (relative to using the same time to harvest from the first patch at a rate $R_{0}$). When averaged over all possible locations $d<d_{S}$ of the second patch, each occurring with probability $P_{1}(d)$, this leads to an expected *gain* in utility (Fig. 2b, right column):
  (3) $$\Delta \langle \bar{U}\rangle_{S3}T=\int_{0}^{d_{S}}2P_{1}(d)\frac{\left|R_{\Delta }\right|}{R_{0}}(d_{L}-d_{S})(R(d)-R_{0})\;dd$$

When planning a sequence of trips, the forager does not know which of these three scenarios it will face, and thus all three scenarios contribute to the expected benefit of exploring the environment in two trips rather than one $\Delta U=\Delta \langle \bar{U}\rangle_{S1}+\Delta \langle \bar{U}\rangle_{S2}+\Delta \langle \bar{U}\rangle_{S3}$ (Fig. 2b and c). If the expected nutritional content is constant across space (i.e. γ=0 and $\hat{R}(d)=\mu >R_{0}$), and in the limit that patches are sparsely distributed (pD≪1), the expected loss in utility from scenario 2 always exceeds the expected gain in utility from scenario 3, regardless of the lengths of the two trips (SI Section 2). As a result, it is always favorable for the forager to explore the environment in a single trip (Fig. 2d; upper left of each heatmap). As the density of patches increases, there is a greater probability of finding food close to the original patch. This reduces the expected loss in utility from scenario 1, and increases the expected gain in utility from scenario 3, thereby encouraging the planning of two trips over one (Fig. 2d; moving from left to right in each heatmap). Similarly, as the expected nutritional content decays more sharply with distance (i.e. as *γ* increases above 0), the expected gain in utility from scenario 3 begins to increasingly outweigh the expected loss from scenario 2, again encouraging the planning of two trips over one (Fig. 2d; moving from top to bottom of each heatmap).

Together, these results show that it becomes increasing beneficial to break a single exploratory trip into a sequence of two trips in environments with a higher density of patches, and in environments where nearby patches are expected to be more nutrient-rich than distant patches (whether because they have higher yield, or whether because it requires less energy to travel between and exploit multiple patches simultaneously).

### Planning for multiple trips can further improve performance

The previous results identified environmental conditions under which it is favorable for the forager to structure its planned exploration in two successive trips rather than one. This suggests that increasing the planning horizon beyond two trips might confer an additional benefit.

**Fig. 3.**
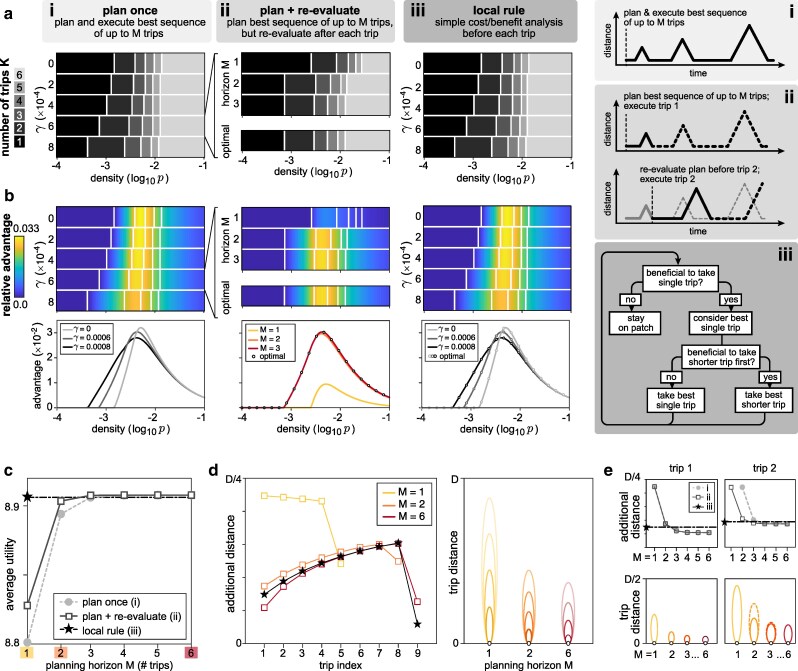
Planning for multiple trips can improve performance. a) Optimal number of trips for different environmental conditions, compared across three different strategies: (i) planning and executing a single optimal trajectory without re-evaluation, (ii) re-evaluating the optimal trajectory after each successive trip, and (iii) implementing a local decision rule on each trip. Strategies (i) and (ii) impose a maximum planning horizon *M*, and determine the best sequence of K≤M trips within that planning horizon. For strategy (i), the planning horizon must equal or exceed the optimal number of trips in order to achieve optimal performance. For strategy (ii), the optimal number of trips can be achieved with a shorter planning horizon. Strategy (iii) achieves optimal performance with a local rule (analogous to a planning horizon of M=1). b) Same as (a), but evaluating the relative advantage (Eq. 4) of taking the optimal sequence of *K* trips compared to best single trip. c) Average utility as a function of planning horizon *M*. Longer planning horizons lead to higher utility; however, the local decision rule can achieve near-optimal utility with only local operations. d) Left: additional distance covered by successive trips for different strategies highlighted in (c). Right: illustration of successive trips highlighted in left panel, split out by different planning horizons. In general, longer planning horizons lead to shorter initial trips; allowing for re-evaluation further changes subsequent trip lengths. e) Additional distance (upper) and trip distance (lower) as a function of planning horizon, shown for first and second trips. The following parameters were used in (a–e): D=1,000, $R_{0}=0.25$, $R_{\Delta }=-0.25$, μ=10, T=24,000; in (c–e): p=0.24771, γ=0.0006.

To explore how expected utility depends on the planning horizon *M*, we numerically solved for the optimal sequence of *K* planned trips $\{d{\}}_{i=1}^{K}$, with $0<d_{1}<d_{2}<\ldots <d_{K}\leq D$, for all values of K=1,2,3,…,M (SI Section 1). We denote $K_{\mathrm{opt}}\leq M$ as the optimal value of *K* that maximizes expected utility (note that in the limit of large *M*, the optimal sequence of $K_{\mathrm{opt}}$ trips is the globally optimal solution, and if $K_{\mathrm{opt}}<M$, increasing *M* does not change the optimal solution). Consistent with the results discussed in the previous section, we find that the optimal number of trips $K_{\mathrm{opt}}$, and thus the optimal planning horizon, increases as the density of resources increases (Fig. 3a-(i)). In other words, resource-rich environments encourage the planning of multiple exploratory trips.

We next measured the relative advantage $s_{\mathrm{opt}}$ of planning and executing the optimal sequence of *K* trips $\{d^{*}{\}}^{K}$ over that of executing the optimal single trip $\{d^{*}{\}}^{1}$:


(4) $$s_{\mathrm{opt}}=\frac{\left\langle U_{\mathrm{mean}}(\{d^{*}{\}}^{K})\rangle -\langle U_{\mathrm{mean}}(\{d^{*}{\}}^{1})\right\rangle }{\left\langle U_{\mathrm{mean}}(\{d^{*}{\}}^{1})\right\rangle }.$$

By computing this relative advantage over a range of environmental conditions, we find that planning for multiple trips confers the greatest advantage when resources are moderately dense (Fig. 3b-(i)). When the density is sufficiently low, the forager is best served by planning a single long trip (as in Fig. 2d). As the density of food patches increases, the likelihood of encountering a new patch increases, making it more favorable to begin exploring earlier using a correspondingly shorter first trip. When the density is sufficiently high, the expected utility of the best single trip is high, and thus the forager gains little by planning any additional trips.

For a given set of environmental conditions, the average utility increases as the planning horizon *M* increases (Fig. 3c), and approaches the globally optimal solution for large *M*. By comparing the planned distances of each trip $\{d{\}}_{i=1}^{K}$ for different planning horizons *M*, we also find that $d_{i}(M)$ decreases with *M* for $i\leq K\leq K_{\mathrm{opt}}$, implying that planning for the possibility of taking multiple trips decreases the planned length of each trip (Fig. 3d).

### Re-evaluation can improve performance but still requires planning for multiple trips

The previous results assume that the forager carries out a planned sequence of trips until it encounters a new food patch, without re-evaluating that plan based on newly acquired information about the environment (namely that explored regions do not contain food). We next asked whether re-evaluation could reduce the optimal planning horizon. To this end, we assume that the forager begins with the same prior beliefs as considered above, but after each successive trip, we allow the forager to update its belief about the location of the nearest patch (note that this belief is only updated when the forager did not encounter a patch during its most recent trip). This updated belief, together with the prior belief about the quality of resources, can then be used to re-evaluate the planned sequence of trips. As before, we assume that the forager determines the best sequence of trips up to a planning horizon of *M* trips, but we now consider the case where the forager takes only the first trip in that sequence. If, on that trip, the forager encounters a food patch at a position $d^{†}$, we assume as before that the forager harvests energy at an average rate $R(d^{†})$ for the remainder of the time *T*. If no new food patch is encountered, the forager returns to the original food patch, updates its belief about the location of the nearest patch, and plans a new sequence of up to *M* trips. The simulation stops when the time exceeds *T*, or when it is no longer favorable to explore (which can occur when the animal has already explored the whole space, or when the remaining unexplored space provides a lower expected energy rate than the original food patch). After a trip to *d_i−1_*, the forager’s belief about the nearest patch is updated to:


(5) $$P_{1}(d_{i})=\left\{ \begin{array}{cc} pe^{-p(d_{i}-d_{i-1})} & \text{for}\;d_{i}\geq d_{i-1}\\ 0 & \text{otherwise}. \end{array}\right.$$

For relatively short planning horizons, this strategy quickly achieves optimal performance, even when the optimal number of trips exceeds the limit of the planning horizon (Fig. 3a-(ii), b-(ii)). Moreover, for a given planning horizon *M*, planning with re-evaluation enables the forager to achieve a higher average utility than planning without re-evaluation (Fig. 3c); equivalently, with re-evaluation, the forager can achieve the same desired utility with a shorter planning horizon. Nevertheless, even with re-evaluation, planning for multiple trips can significantly increase expected utility (Fig. 3c), and is marked by different sequences of trip lengths (Fig. 3d).

One reason that re-evaluation improves performance is that it relaxes the constraint that we otherwise impose on the maximum number of trips that can be carried out in any fixed period of time; in other words, because the forager can re-plan after each trip, the total number of trips is not limited by the planning horizon (Fig. 3d). This is also illustrated in Fig. 3c,e, where the ability to re-evaluate separates the behavior and performance of two foragers with a limited planning horizon of M=1. Both foragers take the same first trip (Fig. 3e, left column), but the forager that re-evaluates takes a second trip (Fig. 3e, right column), which increases its average utility (Fig. 3c). However, the process of re-evaluation still benefits from a longer planning horizon, since the possibility of taking multiple trips can be important for deciding the optimal length of each subsequent trip.

### A local decision strategy can achieve close-to-optimal performance

While iterative planning for multiple trips can improve overall performance, it is computationally costly. We thus asked whether a simpler strategy could achieve comparable performance. To this end, we constructed a two-step local rule for determining whether to leave the food patch, and how far to travel on each successive trip:

**Step 1:** Determine the optimal length of a single trip, $d_{m}^{*}$. If the expected utility of this trip is greater than that of staying on the original food patch, proceed to Step 2; otherwise, continue harvesting from the original patch.
**Step 2:** Weigh the expected benefit and cost of first taking a shorter trip to $d_{s}<d_{m}^{*}$, and determine the optimal value of $d_{s}^{*}$ that maximizes the benefit minus the cost. If the benefit of taking $d_{s}^{*}$ exceeds the cost, plan to travel to $d_{s}^{*}$; otherwise, plan to travel to $d_{m}^{*}$.

Through simulation, we find that this simple local rule achieves near-optimal performance (Fig. 3a-(iii), b-(iii), c).

### The local decision strategy can be implemented in simple neural network architecture

**Fig. 4.**
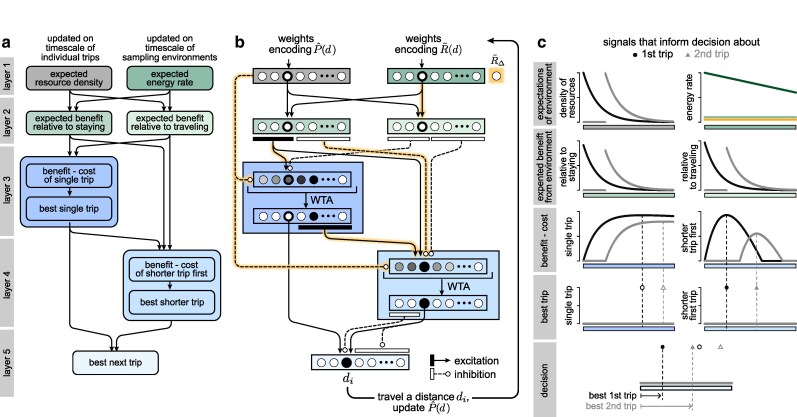
A simple neural network can implement the local decision rule. a) The local decision rule can be broken down into a set of successive computations that are performed in different modules of a neural network, as described in the text and as elaborated in (b). b) A network architecture can implement the local rule using different modules of neurons (colored boxes), each ordered by distance. Weights between modules can be derived from the expressions for the local decision rule, and involve multiple stages of excitatory and inhibitory connections between successive layers. A neuromodulatory signal modulates specific weights (highlighted connections). Here, we show only those weights that involve the third neuron in the network, but the same patterns of weights apply to all neurons in the network. WTA: winner-take-all. c) Example signals in each layer of the network, shown for two successive decisions (trip 1 in black, and trip 2 in gray). The final output in the 5th layer selects the best shortest trip, given the expectations about the environment encoded in the input weights $\hat{P}(d)$ and $\tilde{R}(d)$. We assume that the forager executes the best shortest trip, and updates the weights P(d) accordingly. Shown for D=1000, p=0.0061359, $\tilde{R}(d)=40(1-0.0004d)$, and ${\tilde{R}}_{\Delta }=1$.

Having shown how optimal decisions can be achieved by a simple local decision rule, we now show how this decision rule could be implemented within a simple neural network architecture. This architecture consists of two parallel but interacting streams of operations that are carried out over several feedforward layers with skip connections (Fig. 4a):

- **Layer 1**: encodes signals about expected properties in the environment.
- **Layer 2** [receives inputs from layer 1]: encodes the expected benefit (i.e. increase in energy rate) of encountering a new food patch at each location during foraging relative to staying on the original food patch and relative to traveling.
- **Layer 3** [receives inputs from layers 1 and 2]: computes the relative benefit vs. cost of taking a single trip of any length, and uses the rectify and winner-take-all operations to identify the best single trip, if it exists.
- **Layer 4** [receives inputs from layers 1, 2, and 3]: computes the relative benefit vs. cost of taking a shorter trip given the best single trip, and uses the rectify and winner-take-all operations to identify the best shorter trip (if it exists).
- **Layer 5** [receives inputs from layers 3 and 4]: selects the desired next trip.

We assume that the activity in the first layer is encoded by two sets of input weights: the first set encodes the expected density of food patches (${\vec{w}}_{P}={\hat{P}}_{1}(d)$), and changes over short timescales as the forager actively explores its environment. The second set encodes the expected energetic return of the environment per unit time (relative to the energy rate provided by the original food patch; ${\vec{w}}_{R}=\tilde{R}(d)=\hat{R}(d)/R_{0}$), and changes over longer timescales as the animal learns about the broader environmental statistics.

The remaining computations in layers 2–5 can be performed using a combination of fixed connection weights between layers and fixed operations within layers (Fig. 4b). The effects of energetic constraints enter the circuit through a global signal that encodes the relative energetic cost of travel, ${\tilde{R}}_{\Delta }=|R_{\Delta }|/R_{0}$, and modulates a subset of connection weights accordingly (highlighted connections in Fig. 4b). This modulation allows the same circuit architecture to implement the decision rule under different energetic conditions by adjusting the relevant weights to reflect changes in the relative cost of travel. All required weights and operations can be derived directly from the equations that specify the local decision rule (SI Section 3). These weights impose an ordering over nodes in each layer, such that the activations within each layer can be interpreted as varying as a function of distance from the current food patch. As a result, given a binary input that signals whether or not the forager is on a food patch, the network activity will return the distance that the forager should travel when searching for other food patches (Figs. 4c and S2).

## Discussion

In this work, we investigated the optimal search strategy for an energetically constrained forager that has already discovered a food patch but remains uncertain about the existence of additional patches in the environment. A key assumption of our model is that the forager remembers the location of the known food patch, and reliably returns to it in the event that no new resources are found during exploration. This assumption is motivated by the fact that many animals, including insects such as bees and ants, use internal cues to track their position relative to a reference point (11, 15–17). Such path integration allows them to explore outward from a food source while maintaining the ability to return. Ants, in particular, are well known for their ability to return home along a direct path, even after long and circuitous explorations (17). Crucially, this assumption of spatial memory distinguishes our framework from other foraging models in which movement step lengths are assumed to be drawn from a fixed underlying distribution. For example, in Lévy flight models, step lengths follow a heavy-tailed distribution, which has been shown to yield efficient search performance under certain environmental conditions (8–10). By adopting a prescribed distribution of step lengths, the average statistical structure of search patterns under such models remains constant over time. The classical MVT also typically assumes that a forager departs from one patch and conducts a stochastic, memoryless search for the next one (1). In that setting, the decision to leave is framed as a trade-off between diminishing returns of the current patch and the expected gain from randomly encountered new patches, with no guarantee of return even if no other patches are available.

Other studies of optimal foraging that do take into account spatial memory often posit that the forager knows the locations of all available resources. In such settings, the problem reduces to finding the most efficient route through a set of known targets, often framed as a traveling salesman problem. This formulation has been used to model traplining behavior in pollinators such as bumblebees, which develop stable visitation sequences to known flowers after repeated experience (18, 19 ). Similar behavior has been observed in hummingbirds, which establish repeatable foraging circuits among flower patches (20), and in primates, which revisit productive trees in a consistent order when foraging in familiar areas (21). While models of such behavior address how animals efficiently exploit known environments, our work focuses on how to structure exploratory search before the spatial structure of the environment is known.

Our model provides a framework for analyzing how such decisions should be structured under environmental uncertainty and energetic constraints. By accounting for all possible states of the environment, including whether and where other patches might be present relative to the known food patch, we found that the optimal strategy depends critically on the forager’s prior expectations. When the environment is expected to be sparse, a single long exploratory trip is favored; when patch density is believed to be high and the quality of new patches is expected to decline steeply with distance, it is beneficial to break the exploration into multiple successive trips. This latter pattern, in which foragers make repeated excursions that gradually increase in length, has been observed in *Drosophila* (14); our results offer a normative explanation for why such behavior may be beneficial. While it remains challenging to access animals’ prior beliefs, it is nevertheless possible to shape these beliefs through prior exposure to environments that differ in the density or quality of resources (22). Our results provide concrete predictions for how behavior should differ across these scenarios.

To better understand the computational cost of optimal planning, we derived a local decision rule that approximates the optimal strategy. Although the component calculations involve integrals that may at first appear complex, we showed how these computations can be implemented in neural circuits using fixed connections between populations of neurons, with each population encoding key spatial quantities. The resulting circuit architecture is both conceptually straightforward and biologically plausible, relying on feedforward connectivity and local operations such as additive and multiplicative integration of inputs. As such, our approach aligns with efforts to hand-design biologically plausible circuits for implementing specified computations (23–25), and offers a level of mechanistic and computational interpretability that can be difficult to extract from task-optimized neural networks (26). More broadly, our work also touches on the nature of optimal decision-making in small brains with constrained neural circuits. In many contexts, generating optimal behavior can seem computationally demanding, and might appear to require explicit deliberation, weighing of alternatives, or even conscious reasoning. Yet animals routinely make rapid and seemingly effortless decisions that govern their survival. Our results show how near-optimal strategies can emerge from neural networks that implement local, low-level operations. This suggests that evolution may endow organisms with neural architectures that effectively “solve” complex optimization problems, not through abstract reasoning but through hardwired structure and dynamics.

Planning and decision-making are often studied within the context of Markov decision processes (MPDs), which can be combined with reinforcement learning methods to derive optimal action sequences for maximizing reward. The trip-planning problem that we study can similarly be formulated as an MDP, in which states encode the variables relevant for decision-making (such as the maximum explored range and whether a new food patch has been found within that range), and actions correspond to different choices of trip lengths. In fact, under this mapping, the optimal sequence of trips that maximizes average utility satisfies the Bellman optimality equations (SI Section 1.1). However, we focus here on analyzing the structure and implementation of the optimal strategy given prior beliefs, rather than on modeling how optimal behavior might be learned across repeated episodes, as has been done in the context of patch foraging (27).

To make our problem tractable, we made a number of simplifying assumptions about both the environment and the agent. We considered a 1D and unchanging environment, in which the known food patch does not deplete. We also assumed that the agent has a fixed belief about resource density in unexplored regions, can always return successfully to a remembered location, and consumes sufficient energy between trips to support the next exploratory phase. While these assumptions simplify the analysis, the broader framework is flexible and can be extended to treat a broader range of scenarios. For example, the energy rate provided by the known food patch could decrease with time spent on the patch. In this case, our model suggests that two opposing factors influence the optimal strategy: (i) depletion increases the relative value of discovering alternative patches, thereby favoring earlier or more extensive exploration, but (ii) obtaining enough energy to undertake a given trip requires staying longer on the patch, which delays exploration. The relative strengths of both factors would depend on the specific depletion dynamics. Similarly, if resources can appear over time—such that previously empty regions may later contain resources—foragers that incorporate this possibility into their beliefs would be incentivized to continue exploring even in areas already visited. To allow for such environmental changes as well as initial uncertainty in resource densities, it is beneficial for the forager to maintain and update its beliefs about patch density and other aspects of the environment based on prior experience. Such beliefs would also contain information about the predictability of the environment, which is known to influence animal movement (28). To further account for scenarios where the forager gets lost, trip distances could be modulated by the quality of the forager’s memory and by the ability to successfully execute a return trip; similarly, to account for scenarios where the prior trip was longer or shorter than intended, feeding times could be modulated by prior exploratory bouts. While such modifications introduce additional complexity, the same core approach—solving for the optimal sequence of exploratory decisions to maximize expected utility across a distribution of possible environments, and asking whether the optimal strategy can be achieved using simple rules—remains applicable. Exploring how these extensions affect the structure of the optimal strategy, and whether similarly simple decision rules can be used to approximate optimality, is an interesting direction for future work. These theoretical extensions could also be coupled to future experimental tests. For example, by fixing mean resource density while varying the range of environments to which animals are exposed, one could manipulate the uncertainty in the animals’ prior beliefs. Our framework can then be used to predict how this uncertainty would impact exploratory behavior, and conversely, to infer features of internal beliefs from observed behavior.

More broadly, the framework developed here may be relevant beyond biological foraging. Many real-world scenarios involve exploring uncertain environments under energetic or resource constraints. For instance, autonomous drones and underwater vehicles used in search-and-rescue operations or wildlife monitoring, or planetary rovers exploring the surfaces of celestial bodies, must plan paths that balance the goal of discovering valuable targets with the risk of depleting fuel or battery power before returning to a central base. Similar considerations also arise in virtual environments such as real-time strategy games, where players or artificial agents must plan exploratory actions given limited information about their surroundings. Such settings have become valuable testbeds for studying planning behavior, including recent efforts to develop high-performing artificial agents (29). Even in everyday scenarios, people must structure their search efforts—such as for lost items or online information—in situations where prolonged search might be prohibitively costly and must be broken up into several successive search bouts. This work may therefore offer insights into the structure of optimal search strategies across diverse contexts.

## Supplementary Material

pgag009_Supplementary_Data

## Data Availability

There are no data underlying this work.

## Preprints

This manuscript was posted as a preprint: https://doi.org/10.1101/2025.07.04.663150.
