# Supplementary material for: Efficient planning and implementation of optimal foraging strategies under energetic constraints
Source: PNAS Nexus. 2026 Jan 17;5(2):pgag009. doi: 10.1093/pnasnexus/pgag009 (PMC12888820; doi:10.1093/pnasnexus/pgag009)
Supplement: pgag009_Supplementary_Data [file pgag009_supplementary_data.pdf]

Supplementary Information: Efficient planning and implementation of optimal foraging strategies under energetic constraints

Yipei Guo[1,2†] and Ann M. Hermundstad[1†]

[1]Janelia Research Campus, Howard Hughes Medical Institute, Ashburn, VA, USA
[2]Institute of High Performance Computing, Agency for Science, Technology and Research, Singapore
[†] for correspondence: guo_yipei@a-star.edu.sg, hermundstada@janelia.hhmi.org

1 Expected utility of a planned trajectory

Any given planned trajectory consists of a sequence $\{d\}_i^N$ of N outbound trips to and return trips from locations $d_1, d_2...d_N$. Assuming that the expected quality of a potential new food patch exceeds that of the known patch (as exploration would otherwise not be beneficial), the forager should leave as soon as it has accumulated enough energy for the next trip, since remaining longer reduces the time available to exploit any newly discovered patch. Assuming the forager moves at constant velocity $v = 1$, such that the energy required for a round trip to distance d is $2d|R_\Delta|$, the duration on the patch before the ith trip is therefore given by: $\Omega_i = 2d_i|R_\Delta|/R_0$.

The planned trajectory is the actual trajectory that the animal takes if it does not encounter any new food spot throughout this period, which occurs with probability $p_n = e^{-pd_N}$. In this case, the total time the animal spends away from the original food patch is given by $2d_{\text{tot}} = 2\sum_{i=1}^{R} d_i$, and the corresponding average reward rate $R_{\text{mean,n}}$ of the animal over a time interval $T \geq 2\left(1 + |R_\Delta|/R_0\right) d_{\text{tot}}$ is:

$$\frac{R_{\text{mean,n}}(\{d\}_i^N)}{R_0} = 1 - \left(1 + \tilde{R}_\Delta\right)\frac{2d_{\text{tot}}}{T}, \tag{S1}$$

where $\tilde{R}_\Delta = |R_\Delta|/R_0$. This expression captures the intuition that if no new food patch is found (which must be the case if no other food patch is present), the time spent exploring reduces the animal's average reward rate.

If instead the forager encounters a new food patch at position d, we assume that the reward rate stays at $R(d)$ from then onward (i.e., the forager stops exploring), and the mean reward rate $R_{\text{mean}}(d|\{d\}_i^N)$ over the same time interval T for the planned trajectory is:

$$\frac{R_{\text{mean}}(d|\{d\}_i^N)}{R_0} = \tilde{R}(d) - \left(\tilde{R}(d) - 1\right)\frac{\Omega_{\text{stay}}(d|\{d\}_i^N)}{T} - \left(\tilde{R}(d) + \tilde{R}_\Delta\right)\frac{\Omega_{\text{explore}}(d|\{d\}_i^N)}{T}, \tag{S2}$$

where $\tilde{R}(d) = R(d)/R_0$, and $\Omega_{\text{stay}}(d|\{d\}_i^N)$ and $\Omega_{\text{explore}}(d|\{d\}_i^N)$ are respectively the total time the animal spends on and away from the original food patch before encountering the new food patch.

For any planned trajectory $\{d\}_i^N$, the overall average mean reward rate $\langle R_{\text{mean}}\rangle$, with $\langle\cdot\rangle$ representing the average over the distribution of environments (i.e., potential locations of the nearest food patch), is then given by:

$$\frac{\langle R_{\text{mean}}(\{d\}_i^N)\rangle}{R_0} = p_n\frac{R_{\text{mean,n}}(\{d\}_i^N)}{R_0} + \int p(d)\frac{R_{\text{mean}}(d|\{d\}_i^N)}{R_0}\,\mathrm{d}d. \tag{S3}$$

To obtain the optimal planned trajectory with N trips, we solve for the set of trips $\{d\}_i^N$ that maximizes $\langle R_{\text{mean}}\rangle$.

1.1 Mapping to a Markov decision process

Let $\vec{z} = [z_f, z_e, t_r, n_{\max}]$ denote the agent's current state, which contains all information required for choosing the next trip length that maximizes average utility in the time T after the first encounter with the known food patch (SI Fig. S1a). $z_f \in \{0, 1\}$ indicates whether a new food patch has been found (if $z_f = 0$, the agent is at the original patch; if

$z_f = 1$, the agent is at the new patch), $z_e \in [0, D]$ represents the maximum distance the agent has explored so far, $t_r \in [0, T]$ represents the remaining time left in the environment (that the agent is interested in maximizing its utility over), $n_{\max} \in \{0, 1, 2, ..., M\}$ is the maximum number of future trips the agent is willing to make, with M being the maximum planning horizon. For an agent that follows an optimal sequence of up to M trips without re-evaluating, $n_{\max}$ decreases by 1 after each trip; for an agent that re-evaluates after every trip (with the same planning horizon each time), $n_{\max} = M$ after each trip.

Here, we consider the scenario in which $n_{\max}$ decreases after each trip, and show that finding the policy that maximizes the value of the initial state $\vec{z} = [z_f = 0, z_e = 0, t_r = T, n_{\max} = M]$ corresponds to finding the sequence of up to M trips that maximizes the agent's average utility (Eq. S3).

If $z_f = 1$ or $t_r = 0$ or $n_{\max} = 0$, the agent no longer makes any additional trips. These are therefore the terminal states, and their values $V(\vec{z})$ are given by the total energy gain after the agent has arrived at these states:

$$V(z_f = 1, z_e, t_r, n_{\max}) = R(z_e)t_r \tag{S4}$$
$$V(z_f = 0, z_e, t_r, n_{\max} = 0) = R_0 t_r \tag{S5}$$
$$V(z_f, z_e, t_r = 0, n_{\max}) = 0. \tag{S6}$$

From the non-terminal states $\vec{z} = [z_f = 0, z_e, t_r > 0, n_{\max} > 0]$, the possible actions a are (i) $a = 0$, in which case the agent does not make any next trip, and hence receives reward $r(a = 0|\vec{z}) = R_0 t_r$ and transitions to a terminal state $\vec{z'} = [z'_f = 0, z'_e = z_e, t'_r = 0, n'_{\max}]$ with probability 1, or (ii) $z_e < a < a_{\max}$, in which case the agent makes a trip of length a that is longer than its previously explored range (SI Fig. S1b; note that the maximum allowable length of the next trip $a_{\max}$ is the one for which the agent has sufficient time remaining to accumulate enough energy to undertake).

If the agent makes a next trip (i.e., takes action $a > 0$), there is a probability $e^{-p(a-z_e)}$ that it returns to the original food patch without finding a new patch, in which case it receives no reward (since energy gain from the original patch is fully consumed during the trip) and transitions to state $\vec{z}' = [z'_f = 0, z'_e = a, t'_r = t_r - \Omega(a) - 2a, n'_{\max} = n_{\max} - 1]$ (SI Fig. S1c). If instead the agent discovers a new patch at location z'_e, which occurs with probability $pe^{-p(z'_e - z_e)}$, it receives a reward $r(\vec{z'} = [z'_f = 1, z'_e], a > 0) = R_0\Omega(a) - |R_\Delta| z'_e$ and transitions to state $\vec{z}' = [z'_f = 1, z'_e, t'_r = t_r - \Omega(a) - z'_e]$ (SI Fig. S1a,c).

For any given policy $\Pi(a|\vec{z})$, the value of a state $\vec{z}$ is given by the Bellman equation:

$$V_\Pi(\vec{z}) = \sum_a \Pi(a|\vec{z}) \sum_{\vec{z}'} \sum_r P(\vec{z}', r|\vec{z}, a)\,(r + V_\Pi(\vec{z}'))\,. \tag{S7}$$

For a planned sequence of N trips $[d_1, d_2, ...d_N]$, it is convenient to define the state at the start of the ith trip to be $\vec{z}_{i-1} = [z_f = 0, z_{e,i-1}, t_{r,i-1}, n_{\max,i-1}]$, where $z_{e,j} = d_j$ with $d_0 = 0$, $t_{r,j} = T - \sum_{i=0}^{j}(\Omega_i + 2d_i)$, and $n_{\max,j} = N - j$.

Since the policy is deterministic with $\Pi(a = d_i|\vec{z}_{i-1}) = 1$ and 0 otherwise, and the reward obtained from an action depends only on the state the agent transitions to, the value of each state (under a given sequence of trips) is given by (subtituting the expressions for state transition probabilities, reward and value functions into Eq. S7):

$$V(\vec{z}_{i-1}) = e^{-p(d_i - d_{i-1})}V(\vec{z}_i) + \int_{d_{i-1}}^{d_i} pe^{-p(\tilde{d} - d_{i-1})} \left(R_0\Omega_i - |R_\Delta|\tilde{d} + R(\tilde{d})\left(T - \Omega_{\text{stay}}(\tilde{d}|\{d\}_i^N) - \Omega_{\text{explore}}(\tilde{d}|\{d\}_i^N)\right)\right) \mathrm{d}\tilde{d}, \tag{S8}$$

with $V(\vec{z}_N) = R_0\left(T - \sum_{i=1}^{N}(\Omega_i + 2d_i)\right)$.

By solving Eq. S8 recursively and using the fact that the reward $R_0\Omega_i - |R_\Delta|\tilde{d}$ from discovering a new patch at $\tilde{d}$ can equivalently be expressed as $R_0\Omega_{\text{stay}}(\tilde{d}|\{d\}_i^N) - |R_\Delta|\Omega_{\text{explore}}(\tilde{d}|\{d\}_i^N)$, the value of the initial state can be found to be:

$$V(\vec{z}_0) = \langle R_{\text{mean}}(\{d\}_i^N)\rangle T, \tag{S9}$$

where $\langle R_{\text{mean}}(\{d\}_i^N)\rangle$ is given by Eq. S3.

Therefore, the optimal sequence of trips that maximizes average utility is equivalent to the optimal policy that maximizes the value of the initial state within this Markov decision process.

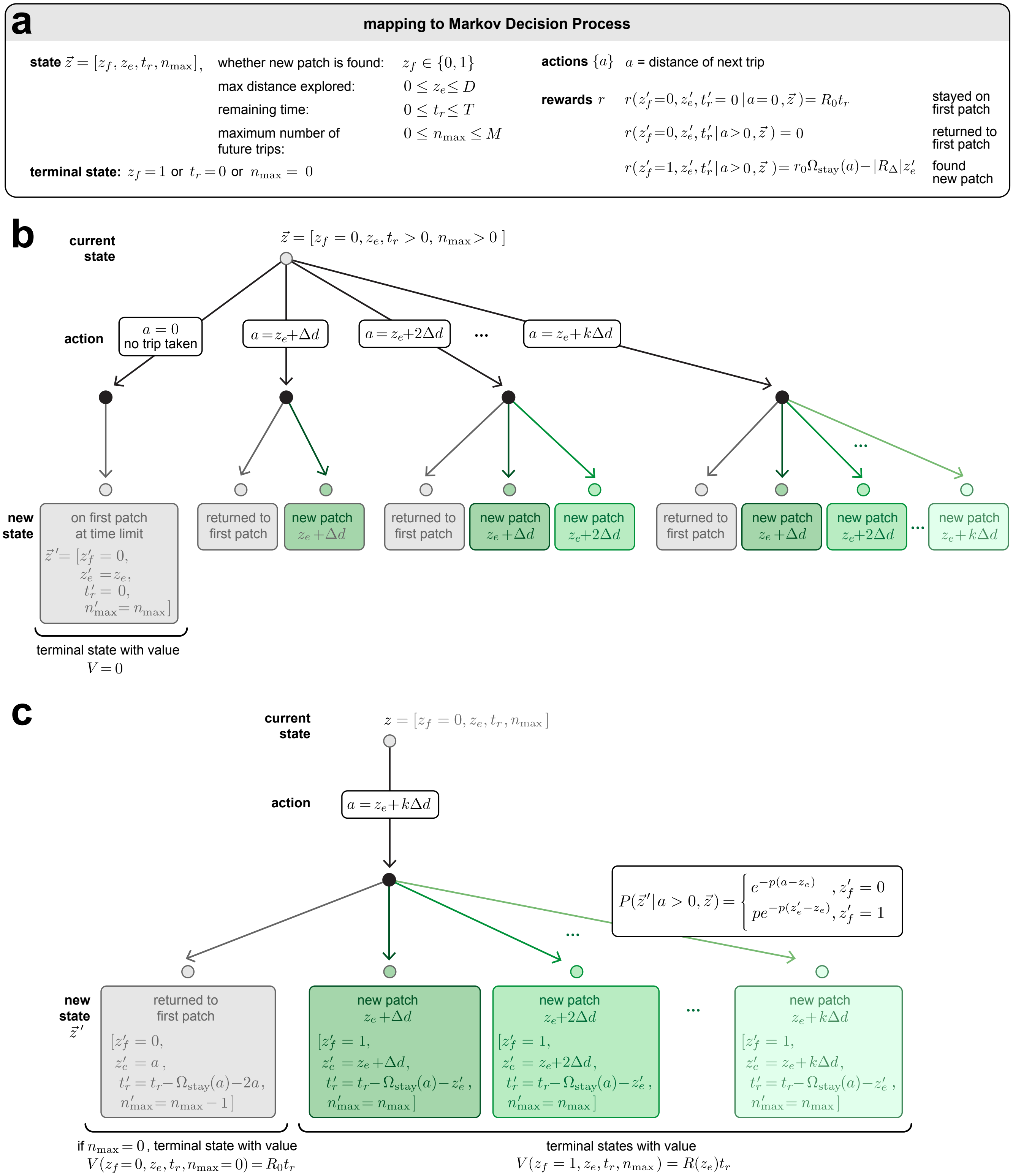

Figure S1: **Mapping to a Markov Decision Process. a)** Summary of key quantities. **b)** Schematic of allowed state transitions for different allowed actions. **c)** Schematic of allowed state transitions for single selected action.

2 Environment with sparse patches and uniform nutritional content

Here, we assume that the nutritional content of patches is constant across space $\hat{R}(d) = \mu > R_0$, and that patches are sparse. In this limit of $pD \ll 1$, the probability that the nearest patch is at location d is given by $P_1(d) = pe^{-pd} \approx p$.

When comparing the utility of taking 2 trips instead of the best single trip, the loss in utility from the scenario where the second patch is far (Scenario 2) is given by (Eq. 2):

$$\begin{aligned}\Delta\langle\bar{U}\rangle_{S2}T &= -\int_{d_S}^{d_L} P_1(d)\left(2d_S(R(d) - R_\Delta) + 2\frac{|R_\Delta|}{R_0}d_S(R(d) - R_0)\right)\mathrm{d}d \\ &\approx -p\left(2d_S(\mu - R_\Delta) + 2\frac{|R_\Delta|}{R_0}d_S(\mu - R_0)\right)(d_L - d_S),\end{aligned} \tag{S10}$$

while the gain in utility from the scenario where the second patch is near (Scenario 3) is given by (Eq. 3):

$$\begin{aligned}\Delta\langle\bar{U}\rangle_{S3}T &= \int_0^{d_S} P_1(d)2\frac{|R_\Delta|}{R_0}(d_L - d_S)(R(d) - R_0)\,\mathrm{d}d \\ &\approx p2\frac{|R_\Delta|}{R_0}(d_L - d_S)(\mu - R_0)d_S \\ &< |\Delta\langle\bar{U}\rangle_{S2}|T\end{aligned} \tag{S11}$$

3 Possible neural circuit implementation of local decision rule

We consider a neural network architecture where there is an indicator neuron that is active when the forager is on the original food patch (and hence has to decide whether and when to leave the food patch and how far to travel on its next trip). The activity of this neuron ($s^I = 1$ when active and $s^I = 0$ when inactive) acts as an input to three sets of neurons in Layer 1 (Fig S2a):

- P**-neurons:** A set of N neurons with input weights $\vec{w}^P = P_1(d)$ representing the probability distribution for the location of the nearest patch.
- $\tilde{R}$**-neurons:** A set of N neurons with input weights $\vec{w}^{\tilde{R}} = R(d)/R_0$ representing the expected energy rate from a potential new patch relative to that from the current patch.
- $\tilde{R}_\delta$**-neuron:** A single neuron with input weights $w^{\tilde{R}_\Delta} = |R_\Delta|/R_0$, representing the ratio between the energy rate expenditure from traveling and the energy rate gain from the current patch.

The belief about the environment, including both the availability of new patches as well as the utility from them, is stored in the weights $\vec{w}^P$ and $\vec{w}^{\tilde{R}}$, and we assume that these can change over time. In particular, $\vec{w}^P$ should be updated as the animal explores and learn about its environment (absence/presence of new food spot), while we expect $\vec{w}^{\tilde{R}}$ and $w^{\tilde{R}_\Delta}$ to be changing over longer timescales. Since the activity of the indicator neuron is binary, the activities for the different neuron types $\alpha \in P, \tilde{R}, \tilde{R}_\Delta$ are also the corresponding input weights when the indicator neuron is active (Fig S2a):

$$\vec{S}^\alpha = \vec{w}^\alpha s^I \tag{S12}$$

With this, we then ask how the output activities of these Layer 1 neurons can serve as inputs to downstream neurons to determine the desired next trip length and the feeding duration on the current patch before leaving.

3.1 How far to travel

Recall that the two-step local rule for determining whether to leave the food patch, and how far to travel on each successive trip is as follows:

Step 1: Determine the optimal length of a single trip, d_m^*. If the expected utility of this trip is greater than that of staying on the original food patch, proceed to Step 2; otherwise, stay on the original patch and continue feeding.

Step 2: Weigh the expected benefit and cost of first taking a shorter trip to $d_1 < d_m^*$ (assuming that if no new patch is found on the trip to d_m^*, the forager continues exploring the remaining space), and determine the optimal value of d_1^* that maximizes the benefit minus the cost. If the benefit of taking d_1^* exceeds the cost, plan to travel to d_1^*; otherwise, plan to travel to d_m^*.

To construct a circuit that takes inputs from the Layer 1 neurons and outputs a representation of the next trip distance (either 0 or d_m^* or d_1^*), it is therefore useful to break down the computations that are required for each step.

Step 1:

From Eq. S3, the relative advantage of making a single-trip trajectory to d_m compared to remaining on the food original patch for a period of time T is given by:

$$\begin{aligned} s_{\text{single trip}}(d_m) = \frac{\langle U(d_m)\rangle}{R_0} - 1 = -2\left(1+\tilde{R}_\Delta\right)\frac{d_m}{T}\left(1-\int_0^{d_m} P_1(d)\,\mathrm{d}d\right) \\ + \int_0^{d_m} P_1(d)\left((\tilde{R}(d)-1)\left(1-2\tilde{R}_\Delta\frac{d_m}{T}\right)-(\tilde{R}(d)+\tilde{R}_\Delta)\frac{d}{T}\right)\mathrm{d}d. \end{aligned} \tag{S13}$$

To carry out this computation, it is useful to have two sets of N neurons in a downstream layer (Layer 2), the activities of which are given by (Fig S2b):

$$S_1\textbf{-neurons:} \qquad \vec{S}_1 = \vec{S}^P \cdot (\vec{S}^{\tilde{R}} - 1) \tag{S14}$$

$$S_2\textbf{-neurons:} \qquad \vec{S}_2 = \vec{S}^P \cdot (\vec{S}^{\tilde{R}} + S^{\tilde{R}_\delta}). \tag{S15}$$

This can be achieved if each neuron $i = 1, 2, ..., N$ in these sets receives inputs from the corresponding i^{th} P- and $\tilde{R}$- neurons, as well as from the $\tilde{R}_\Delta$ neuron in the case of the S_2-neurons. The signal from the $\tilde{R}$- neuron is either offset by 1 (for the S_1-neurons) or summed with the activity of the $\tilde{R}_\Delta$ neuron (for the S_2-neurons), before combining multiplicatively with the activity from the P-neuron.

We then consider another set of N neurons ('d_m-neurons') in a downstream layer (Layer 3) that receives inputs from both Layer 1 (P- and $\tilde{R}$- neurons) and Layer 2 (S_1-, S_2- neurons), such that the overall external inputs $\vec{I}^{d_m}$ to these d_m-neurons are given by (Fig S2b):

$$\begin{aligned} \vec{I}^{d_m} &= \max(\mathbf{W}^{S_1\to d_m}\vec{S}_1 - \mathbf{W}^{S_2\to d_m}\vec{S}_2 + \vec{b}^{d_m}, 0) \\ &= \max(s_{1trip}(d_m), 0) \end{aligned} \tag{S16}$$

where the weights $W_{ij}^{S_1\to d_m}$ are the connection strengths from the jth S_1- neuron to i^{th} d_m- neuron, $W_{ij}^{S_2\to d_m}$ are the connection strengths from the jth S_2- neuron to i^{th} d_m- neuron, and $\vec{b}^{d_m}$ is the bias term that depends only on inputs from Layer 1 (namely, the P- and $\tilde{R}_\Delta$- neurons).

Based on the expression for $s_{1trip}(d_m)$ (Eq. S13), the elements of these weights and bias terms are given by:

$$W_{ij}^{S_1\to d_m} = \begin{cases} (1-2\tilde{R}_\Delta\frac{i}{T})\delta d & \text{if } j \le i \\ 0 & \text{otherwise} \end{cases} \tag{S17}$$

$$W_{ij}^{S_2\to d_m} = \begin{cases} \frac{j}{T}\delta d & \text{if } j \le i \\ 0 & \text{otherwise} \end{cases} \tag{S18}$$

$$b_i^{d_m} = -2(1+\tilde{R}_\Delta)\frac{i\delta d}{T}\left(1-\delta d\sum_{j\le i} S_j^P\right), \tag{S19}$$

where δd is the spatial resolution. It is useful to note that the structure of these input connections is fixed and encodes the spatial geometry and structure of the problem. For some of these connections (namely those from the S_1- and P- neurons), their connection strengths are modulated by $\tilde{R}_\Delta$, suggesting that the activity of the $\tilde{R}_\Delta-$ neuron serves as a global signal for the whole network. Therefore, it might also be useful for the value of $\tilde{R}_\Delta$ to be encoded by certain chemical/hormone levels, and one can imagine the activity of the $\tilde{R}_\Delta$- neuron regulating the release of such chemicals/hormones.

One could then imagine additional connections among the d_m- neurons that implement a winner-take-all operation, such that only the neuron that receives the highest input will remain active (Fig S2b), and the identity of this active neuron encodes d_m^*. In other words, the activities $\vec{S}^{d_m}$ of these neurons are given by:

$$S_i^{d_m} = \begin{cases} 1 & \text{if } I_i^{d_m} > 0 \text{ and } i = \underset{j}{argmax}\left(I_j^{d_m}\right) \\ 0 & \text{otherwise.} \end{cases} \tag{S20}$$

This representation of d_m^* can then be used to determine whether it is advantageous to take a shorter trip first.

Step 2:

Analogous to the d_m- neurons that provide a representation of the value of d_m^*, we assume that there exists another set of N neurons('d_1- neurons') whose binary activities $\vec{S}^{d_1}$ encodes the best shorter trip d_1^* (if it exists). This can be achieved if the net inputs $\vec{I}^{d_1}$ to these neurons are given by the net benefit of traveling to d_1 (if positive, and zero otherwise), and additional connections among the d_1- neurons implement a winner-take-all operation that result in at most one of those neurons being active (Fig S2c):

$$S_i^{d_1} = \begin{cases} 1 & \text{if } I_i^{d_1} > 0 \text{ and } i = \underset{j}{argmax}\left(I_j^{d_1}\right) \\ 0 & \text{otherwise.} \end{cases} \tag{S21}$$

To infer the network connections (between the upstream neurons in Layers 1-3 and these d_1- neurons) needed to achieve the desired inputs $\vec{I}^{d_1}$, we return to the computations required.

From Eqs. 1-3, and assuming that the forager eventually explores the whole space (of length D) if no new patches are found, the net benefit (expected gain in average energy rate) of first taking a short trip to $d_1 < d_m^*$ relative to the baseline energy rate of staying on the current food patch is given by:

$$\begin{aligned} \frac{\Delta U(d_1|d_m^*)}{R_0} &= 2\tilde{R}_\Delta(d_m^* - d_1)\int_0^{d_1} P_1(d)(\tilde{R}(d) - 1)\,\mathrm{d}d \\ &- \left(2d_1\int_{d_1}^{D} P_1(d)\left(\tilde{R}_\Delta(\tilde{R}(d) - 1) + (\tilde{R}(d) + \tilde{R}_\Delta)\right)\mathrm{d}d + e^{-pL}2d_1(1 + \tilde{R}_\Delta)\right). \end{aligned} \tag{S22}$$

The desired inputs to the d_1- neurons can therefore be expressed as:

$$\vec{I}^{d_1} = \max\left(\left((\mathbf{W}^{d_m\to d_1}\vec{S}^{d_m}) \odot (\mathbf{W}^{S_1\to d_1,\mathrm{ext}}\vec{S}_1) - (\mathbf{W}^{S_1\to d_1,\mathrm{inh}}\vec{S}_1) - (\mathbf{W}^{S_2\to d_1,\mathrm{inh}}\vec{S}_2)\right) + \vec{b}^{d_1}, 0\right), \tag{S23}$$

where the weights from the d_m neurons $\mathbf{W}^{d_m\to d_1}$ are:

$$W_{ij}^{d_m\to d_1} = \begin{cases} 2\tilde{R}_\Delta(j-i)\delta d & \text{if } j > i \\ 0 & \text{otherwise,} \end{cases} \tag{S24}$$

the weights of the activating connections $\mathbf{W}^{S_1\to d_1,\mathrm{act}}$ from the S_1- neurons are:

$$W_{ij}^{S_1\to d_1,\mathrm{act}} = \begin{cases} \delta d & \text{if } j \leq i \\ 0 & \text{otherwise,} \end{cases} \tag{S25}$$

the weights of the inhibitory connections $\mathbf{W}^{S_1\to d_1,\mathrm{inh}}$ from the S_1- neurons are:

$$W_{ij}^{S_1\to d_1,\mathrm{inh}} = \begin{cases} 2\tilde{R}_\Delta i\delta d & \text{if } j > i \\ 0 & \text{otherwise,} \end{cases} \tag{S26}$$

the weights of the inhibitory connections $\mathbf{W}^{S_2\to d_1,\mathrm{inh}}$ from the S_2- neurons are:

$$W_{ij}^{S_2\to d_1,\mathrm{inh}} = \begin{cases} 2i\delta d & \text{if } j > i \\ 0 & \text{otherwise,} \end{cases} \tag{S27}$$

and the bias terms $\vec{b}^{d_1}$ (that depend only on inputs from Layer 1) are given by:

$$b_i^{d_1} = -2i\delta d(1 + \tilde{R}_\Delta)\left(1 - \delta d \sum_j S_j^P\right). \tag{S28}$$

As for the input connections to the d_m- neurons, these input connections to the d_1- neurons also encode the spatial geometry and structure of the problem, with some of the weights modulated either directly or indirectly by the $\tilde{R}_\Delta$-neuron.

Finally, a set of N neurons ('d_{next}-neurons') receives inputs from both the d_m and d_1 neurons to provide a representation of the desired next trip distance (Fig S2d). The input weights to the ith d_{next}-neuron are given by:

$$W_{ij}^{d_m \to d_{\text{next}}} = \begin{cases} 1 & \text{if } j = i \\ 0 & \text{otherwise} \end{cases} \tag{S29}$$

$$W_{ij}^{d_1 \to d_{\text{next}}} = \begin{cases} 1 & \text{if } j = i \\ -1 & \text{if } j < i \\ 0 & \text{otherwise} \end{cases} \tag{S30}$$

such that the activities of the d_{next}-neurons are given by:

$$\vec{S}^{d_{\text{next}}} = \mathbf{W}^{d_m \to d_{\text{next}}}\vec{S}^{d_m} + \mathbf{W}^{d_1 \to d_{\text{next}}}\vec{S}^{d_1} \tag{S31}$$

$$\implies S_i^{d_{\text{next}}} = \begin{cases} 1 & \text{if } x_i = d_1^* \text{ (if it exists) or } d_m^* \text{ (if it exists but } d_1^* \text{ does not)} \\ 0 & \text{otherwise} \end{cases} \tag{S32}$$

3.2 How long to stay on food patch before leaving

The set of d_{next}-neurons that encode how far the forager wants to go on its next trip and another set of neurons (e.g. place cells) that encode the current location of the forager, together with the global signal $\tilde{R}_\Delta$, can serve as inputs to a downstream neuron to compute the duration on the food patch before leaving $\Omega(d) = 2\tilde{R}_\Delta d$.

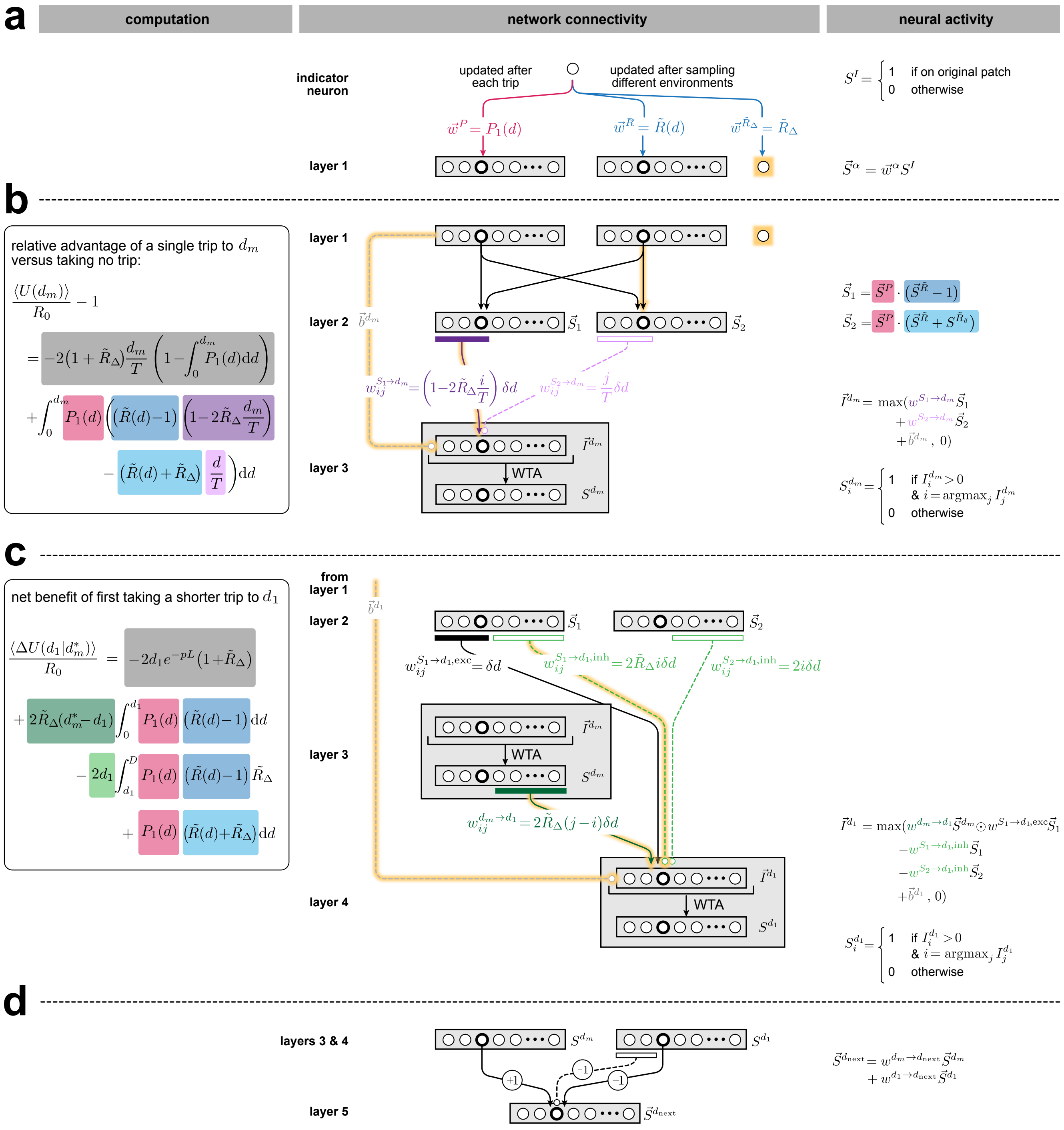

Figure S2: **Breakdown of neural network that implements the local decision rule. a)** Schematic of an indicator neuron (representing whether the forager is on the original food patch) and how it is connected to Layer 1 neurons that encode properties of the environment. **b)** Schematic showing how computations of the relative advantage of a single trip compared to remaining on the original food patch (step 1 of the local rule) can be implemented in the circuit using additional layers of neurons. **c)** Schematic showing how computations of the net benefit of taking a shorter trip first (step 2 of the local rule) can be implemented in the circuit using an additional set of d_1 neurons that encodes the best shorter trip. **d)** Schematic showing how d_m and d_1 neurons can provide inputs to a final set of d_{next} neurons to provide a representation of how far the forager should go next.
